
JOURNAL INFORMATION
==============================
NLM Title Abbreviation: J Diabetes Investig
Journal ID: J Diabetes Investig
NLM Journal ID: 101520702
Journal ID: JDI

Journal of Diabetes Investigation

ISSN: 2040-1116
EISSN: 2040-1124
Publisher: Wiley

ARTICLE INFORMATION
==============================
PMCID: PMC11474572
DOI: 10.1111/jdi.14217
Article ID: JDI14217
Article version: 1
Subjects: Issue Information, Issue Information

Issue Information

Electronic publication date: 2024 Apr 30
Publication date: 2024 May
Volume: 15
Issue: 5
First page: 519
Last page: 520
Copyright: © 2024 The Authors. Journal of Diabetes Investigation published by Asian Association for the Study of Diabetes (AASD) and John Wiley & Sons Australia, Ltd
License: This is an open access article under the terms of the http://creativecommons.org/licenses/by/4.0/ License, which permits use, distribution and reproduction in any medium, provided the original work is properly cited.
License URL: https://creativecommons.org/licenses/by/4.0/

Figure count: 0
Table count: 0
Page count: 5

source-schema-version-number: 2.0
cover-date: May 2024
details-of-publishers-convertor: Converter:WILEY_ML3GV2_TO_JATSPMC version:6.4.9 mode:remove_FC converted:15.10.2024

==============================

### JDI

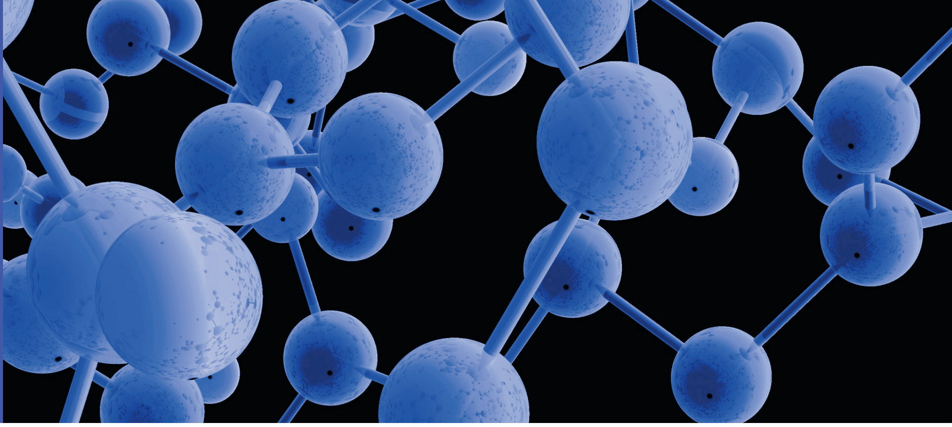

Volume 15 | Issue 5
May 2024

### Journal of Diabetes Investigation

Official Journal of the Asian Association for the Study of Diabetes

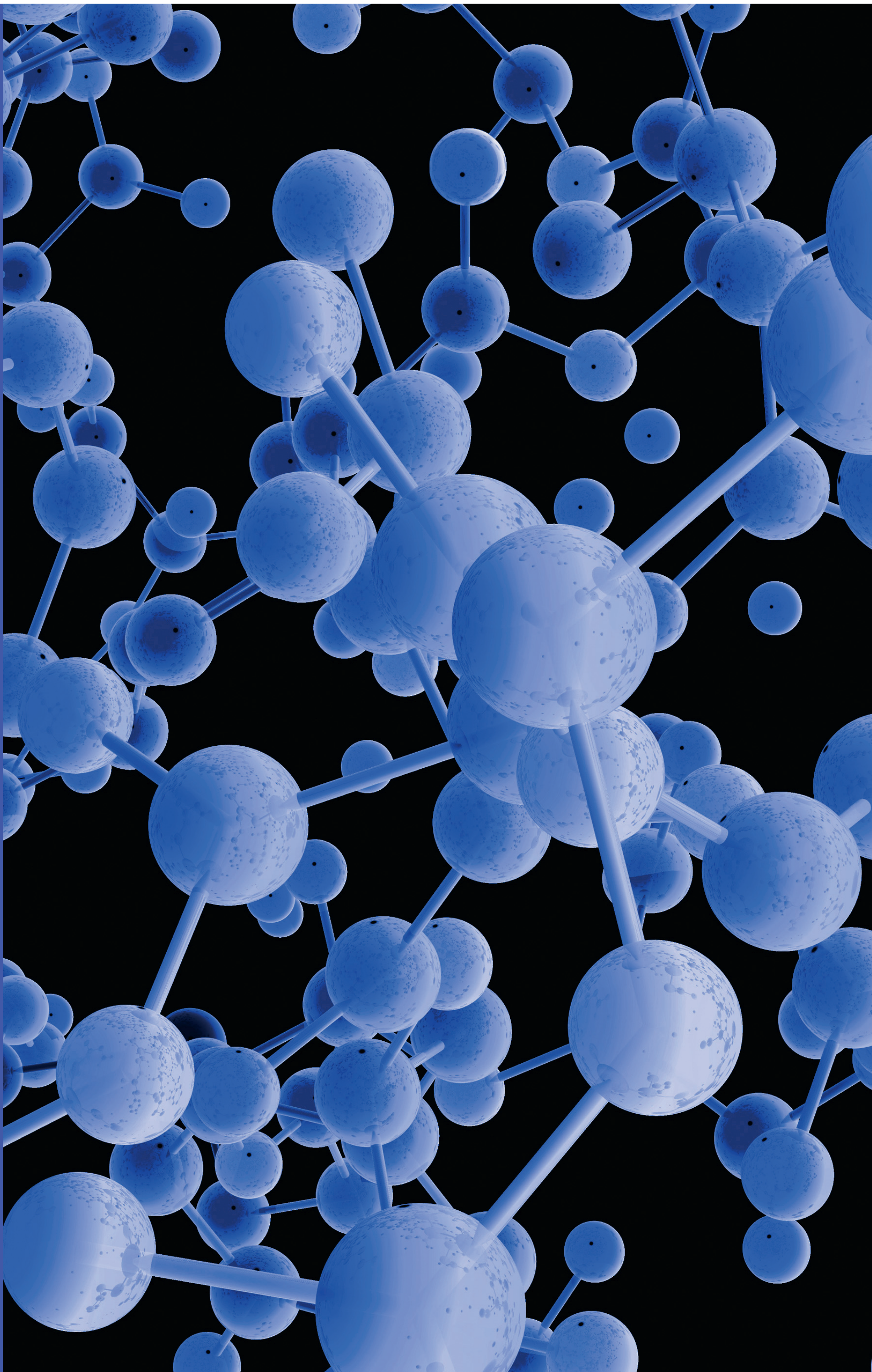

Insulin sensitivity online calculator

THE ASIAN ASSOCIATION FOR THE STUDY OF DIABETES
AASD

WILEY

**JDI Journal of Diabetes Investigation**

Open access

**Official Journal of the Asian Association for the Study of Diabetes**

**Editor-in-Chief**
Nigishi Hotta, *Japan Organization of Occupational Health and Safety, Chubu Rosai Hospital, Nagoya, Japan*

**Co-Editor**
Domenico Accili, *Columbia University, New York, USA*

**Expert Editor**
Bo Ahren, *Lund, Sweden*
Young Min Cho, *Seoul, Korea*
Sean Dinneen, *Galway, Ireland*
Marc Gilbert, *Montpelier, France*
Limei Liu, *Shanghai, China*
Ronald Ma, *Hong Kong, China*
Louis Philipson, *Chicago, USA*
Wayne H-H Sheu, *Taichung, Taiwan*
Bernard Thorens, *Lausanne, Switzerland*
Daisuke Yabe, *Gifu, Japan*

**Editorial Board**
Eiichi Araki, *Kumamoto, Japan*
Xiaoping Chen, *Beijing, China*
Lee-Ming Chuang, *Taipei, Taiwan*
Masakazu Haneda, *Asahikawa, Japan*
Larry Low-Tone Ho, *Taipei, Taiwan*
Tianpei Hong, *Beijing, China*
Akihisa Imagawa, *Osaka, Japan*
Nobuya Inagaki, *Kyoto, Japan*
Linong Ji, *Beijing, China*
Weiping Jia, *Shanghai, China*
Yi-Der Jiang, *Taipei, Taiwan*
Takashi Kadowaki, *Tokyo, Japan*
Kohei Kaku, *Kurashiki, Japan*
Keizo Kanasaki, *Izumo, Japan*
Doo-Man Kim, *Seoul, Korea*
Karen Lam, *Hong Kong, China*
Hong Kyu Lee, *Gyeonggi-do, Korea*
In-Kyu Lee, *Daegu, Korea*
Moon-Kyu Lee, *Seoul, Korea*
Jiro Nakamura, *Nagakute, Japan*
Kishio Nanjo, *Wakayama, Japan*
Wataru Ogawa, *Kobe, Japan*
Yutaka Seino, *Osaka, Japan*
Shyi-Jang Shin, *Kaohsiung, Taiwan*
Hirohito Sone, *Niigata, Japan*
Kathryn Tan, *Hong Kong, China*
Yukio Tanizawa, *Ube, Japan*
Kohjiro Ueki, *Tokyo, Japan*
Jun Wada, *Okayama, Japan*
Hirotaka Watada, *Tokyo, Japan*
Jianping Weng, *Guangzhou, China*
Kyu-Chang Won, *Daegu, Korea*
Soroku Yagihashi, *Hirosaki, Japan*
Hiroshi Yamamoto, *Kanazawa, Japan*
Toshimasa Yamauchi, *Tokyo, Japan*
Wenying Yang, *Beijing, China*
Kazuki Yasuda, *Tokyo, Japan*
Koutaro Yokote, *Chiba, Japan*
Kun-Ho Yoon, *Seoul, Korea*
Dalong Zhu, *Nanjing, China*

**International Editorial Board**
Michael A. Brownlee, *New York, USA*
Subrata Chakrabarti, *Ontario, Canada*
Mark E. Cooper, *Prahran, Australia*
Philippe Froguel, *London, UK*
Wilfred Fujimoto, *Hawaii, USA*
Michael S. German, *San Francisco, USA*
Andrew Hattersley, *Exeter, UK*
Jean-Claude Henquin, *Brussels, Belgium*
Jens Juul Holst, *Copenhagen, Denmark*
Frank B. Hu, *Boston, USA*
C. Ronald Kahn, *Boston, USA*
George L. King, *Boston, USA*
Rayaz A. Malik, *Manchester, UK*
Michael Mauer, *Minneapolis, USA*
Carl Erik Mogensen, *Aarhus, Denmark*
Steven E. Shoelson, *Boston, USA*
Jay S. Skyler, *Miami, USA*
Paul Zimmet, *Melbourne, Australia*

**Assistant Editorial Board**
Tien-Jyun Chang, *Taipei, Taiwan*
Yoshiyuki Hamamoto, *Osaka, Japan*
Norio Harada, *Kyoto, Japan*
Yi-Jen Hung, *Taipei, Taiwan*
Chi-Min Hwu, *Taipei, Taiwan*
Hideki Kamiya, *Aichi, Japan*
Dae Jung Kim, *Suwon, Korea*
Seung-Hwan Lee, *Seoul, Korea*
Hung-Yuan Li, *Taipei, Taiwan*
Takashi Matsuzaka, *Tsukuba, Japan*
Keiko Naruse, *Aichi, Japan*
Takeshi Nishikawa, *Kumamoto, Japan*
Eung-Jung Rhee, *Seoul, Korea*
Yusuke Seino, *Aichi, Japan*
Hironori Waki, *Tokyo, Japan*

**Aims and Scope:** *Journal of Diabetes Investigation* is your core diabetes journal from Asia; the official journal of the Asian Association for the Study of Diabetes (AASD). The journal publishes original research, country reports, commentaries, reviews, mini-reviews, case reports, letters, as well as editorials and news. The journal embraces clinical and experimental research in diabetes and related areas. This includes aspects of prevention, treatment, as well as molecular aspects and pathophysiology. Translational research focused on the exchange of ideas between clinicians and researchers is also welcome.

**Cover:** Insulin molecule © Pasieka/Science Photo Library

This journal is available online at Wiley Online Library. Visit wileyonlinelibrary.com to search the articles and register for table of contents and e-mail alerts.

**For submission instructions, and all other information visit wileyonlinelibrary.com/journal/jdi**

JDI.JEB.May24

**Open Access and Copyright**
All articles published by *Journal of Diabetes Investigation* are fully open access: immediately freely available to read, download and share. All *Journal of Diabetes Investigation* articles accepted from 24 September 2013 are published under the terms of the Creative Commons Attribution 4.0 Unported (CC BY 4.0) license, which permits use, distribution and reproduction in any medium, provided the original work is properly cited. Articles accepted before this date were published under the agreement as stated in the final article. Copyright on any *Journal of Diabetes Investigation* article accepted after 24 September 2013, the date on which *Journal of Diabetes Investigation* became an open access journal, is retained by the author(s). Authors grant to *Journal of Diabetes Investigation* and Wiley a license to publish the article and for Wiley to identify itself as the original publisher. Authors also grant any third party the right to use the article freely as long as its integrity is maintained and its original authors, citation details and publisher are identified. Further information about open access licenses and copyright can be found at: https://authorservices.wiley.com/author-resources/Journal-Authors/licensing/licensing-info-faqs.html#4. Information on the licenses used by Journal of Diabetes Investigation can be found at: wileyonlinelibrary.com/journal/jdi

**Disclaimer**
The Publisher, the Asian Association for the Study of Diabetes, and Editors cannot be held responsible for any errors in or any consequences arising from the use of information contained in this journal. The views and opinions expressed do not necessarily reflect those of the Publisher, the Asian Association for the Study of Diabetes or Editors, neither does the publication of advertisements constitute any endorsement by the Publisher, the Asian Association for the Study of Diabetes, Editors, or Authors, of the products advertised.

Wiley Open Access articles posted to repositories or websites are without warranty from Wiley of any kind, either express or implied, including, but not limited to, warranties of merchantability, fitness for a particular purpose, or non-infringement. To the fullest extent permitted by law Wiley disclaims all liability for any loss or damage arising out of, or in connection, with the use of or inability to use the content.

JDI.PI.May24

VOLUME 15 – ISSUE 5 – MAY 2024

### Journal of Diabetes Investigation

Open access

### Editorial

**Common metabolite for microangiopathy in Japanese and European populations**
Nobuhiro Shojima and Toshimasa Yamauchi 523

### Review

**Cancer biology in diabetes update: Focusing on antidiabetic drugs**
Emi Kawakita and Keizo Kanasaki 525

### Mini Review

**Social deprivation and diabetic kidney disease: A European view**
Caoimhe Casey, Claire M Buckley, Patricia M Kearney, Matthew D Griffin, Sean F Dinneen, and Tomás P Griffin 541

### Commentary

**Birthweight and risk of diabetes in later life**
Wayne Huey-Herng Sheu 557

### Original Articles

### Basic Science and Research

**Oxidative stress suppresses PHB2-mediated mitophagy in β-cells *via* the Nrf2/PHB2 pathway**
Shan Liu, Rui Zhang, Lan Zhang, Aige Yang, Yuqing Guo, Lei Jiang, Huijuan Wang, Shunjiang Xu, and Huimin Zhou 559

**Immunoglobulin heavy constant gamma 1 silencing decreases tonicity-responsive enhancer-binding protein expression to alleviate diabetic nephropathy**
Qibo Hu, Qingxiao Yang, Hang Gao, Jing Tian, and Guanghua Che 572

**Microvascular permeability and texture analysis of bone marrow in diabetic rabbits with critical limb ischemia based on dynamic contrast-enhanced magnetic resonance imaging**
Liu Yang, Donghang Li, Yuchen Yan, Qi Yang, Liang Li, and Yunfei Zha 584

### Short Report

### Clinical Science and Care

**Interactive calculator to estimate insulin sensitivity in type 1 diabetes**
Andrzej S Januszewski, Pawel Niedzwiecki, Nirupa Sachithanandan, Glenn M Ward, David N O'Neal, Dorota A Zozulinska-Ziolkiewicz, Aleksandra A Uruska, and Alicia J Jenkins 594

### Original Articles

**Comparison of efficacy and safety of insulin degludec/liraglutide and insulin glargine U-100/lixisenatide in individuals with type 2 diabetes mellitus using professional continuous glucose monitoring**
Yuji Kawaguchi, Yuriko Hajika, Maho Rinka, Koji Masumoto, Jun Sawa, Kenji Hamazaki, and Yasuro Kumeda 598

**The correlation between serum asprosin and left ventricular diastolic dysfunction in elderly patients with type 2 diabetes mellitus in the community**
Dong Liang, Guoliang Shi, Mingang Xu, Jianhong Yin, Yunfeng Liu, Jing Yang, and Linxin Xu 608

**Association between glycemia risk index and arterial stiffness in type 2 diabetes**
Lingli Cai, Wenqi Shen, Jiang Li, Bin Wang, Ying Sun, Yi Chen, Ling Gao, Fei Xu, Xinhua Xiao, Ningjian Wang, and Yingli Lu **614**

**Type 1 and type 2 diabetes mortality burden: Predictions for 2030 based on Bayesian age-period-cohort analysis of China and global mortality burden from 1990 to 2019**
Chunping Dong, Guifu Wu, Hui Li, Yuan Qiao, and Shan Gao **623**

**Elevated small dense low-density lipoprotein cholesterol to high-density lipoprotein cholesterol ratio is associated with an increased risk of metabolic dysfunction associated fatty liver disease in Chinese patients with type 2 diabetes mellitus**
Shouxing Yang and Jing Xu **634**

### Correction

**Correction to New diagnostic criteria (2023) for slowly progressive type 1 diabetes (SPIDDM): Report from Committee on Type 1 Diabetes of the Japan Diabetes Society (English version)** **643**
